# An unusual cause of a vesical mass: Small bowel diverticulum presenting with a bladder fistula: A case report and review of the literature

**DOI:** 10.1097/MD.0000000000048536

**Published:** 2026-04-24

**Authors:** Jinting Wu, Yinglei Wang, Yang Zhao, Dongbing Zheng, Guangming Shan

**Affiliations:** a Department of Urology, Binzhou Medical University Yantai Affiliated Hospital, Medical Graduate School, Binzhou Medical University, Yantai, China; b The Second Ward of Urology, Binzhou Medical University Yantai Affiliated Hospital, Yantai, China.

**Keywords:** bladder mass, case report, diagnostic challenge, small bowel diverticulum, vesico-enteric fistula

## Abstract

**Rationale:**

Small intestinal diverticulum is rare (<5% incidence) and often asymptomatic. Its presentation as a bladder mass mimicking tumor, leading to vesico-enteric fistula, is exceptionally uncommon. This case highlights the diagnostic challenge and potential for misdiagnosis.

**Patient concerns:**

A 35-year-old woman presented with a complex clinical course spanning 4 surgical interventions over 20 months. Initial symptoms included urinary frequency, urgency, dysuria, and persistent right lower quadrant abdominal pain. Recurrent “bladder tumors” were noted on serial cystoscopies, and the patient experienced expulsion of flocculent, particulate matter during micturition postoperatively.

**Diagnosis:**

Dynamic cystoscopy revealed a mass protruding through a bladder wall defect when the bladder was empty and retracting when filled. Laparoscopic exploration confirmed a 3 × 3 cm small bowel diverticulum located 20 cm proximal to the ileocecal valve, adherent to the bladder with a 1 × 1 cm fistula. Histopathology showed chronic inflammation of the diverticulum and reactive nephrogenic adenoma-like hyperplasia in the bladder mucosa, with no evidence of malignancy.

**Interventions:**

The patient underwent combined laparoscopic diverticulectomy and two-layer bladder fistula repair with omental flap interposition. Intraoperative frozen section of the bladder edge confirmed chronic inflammation with glandular metaplasia, negative for malignancy.

**Outcomes:**

Postoperative recovery was uneventful. At 3-month follow-up, cystoscopy showed a healed fistula site with no recurrence, and computed tomography enterography revealed no residual diverticulum or fistula. The patient’s symptoms completely resolved.

**Lessons:**

Small bowel diverticulum can masquerade as recurrent bladder tumor over multiple surgeries. Key diagnostic clues include the dynamic protrusion/retraction phenomenon with bladder filling status and flocculent material in urine. Persistent “recurrent” bladder masses with atypical features warrant consideration of enteric sources. Multidisciplinary collaboration between urology and general surgery was essential for definitive diagnosis and management.

## 1. Introduction

Small intestinal diverticula are uncommon clinical entities, with an estimated prevalence of approximately 5%.^[1]^ Most cases remain asymptomatic and are incidentally discovered during imaging or surgical procedures for unrelated conditions.^[2]^ However, complications such as diverticulitis, perforation, obstruction, or fistula formation may occur, leading to nonspecific symptoms that mimic common abdominal pathologies.^[3]^ The preoperative diagnosis of complicated small bowel diverticula is exceptionally challenging, with misdiagnosis rates exceeding 90% in some cohorts.^[4]^ This diagnostic difficulty stems from 3 key factors: lack of specific clinical manifestations:symptoms (e.g., abdominal pain, bleeding, and urinary changes) often overlap with appendicitis, inflammatory bowel disease, or tumors, causing clinicians to prioritize common diagnoses over rare entities.^[5]^ Limitations of routine examinations: standard imaging (computed tomography [CT], ultrasound) and endoscopy frequently fail to identify diverticular structures, especially when complications obscure anatomical details.^[6]^ For instance, bladder involvement may present as “recurrent tumors” on cystoscopy, while fistulae manifest as atypical urinary symptoms (e.g., flocculent material excretion).^[7]^ Cognitive bias in clinical decision-making: prior diagnostic labels (e.g., “malignant bladder tumor”) often anchor subsequent evaluations, delaying consideration of alternative etiologies.^[8]^

The current case presents an unprecedented diagnostic odyssey: a 35-year-old woman underwent 4 surgeries between 2023 and 2024 for a lesion initially misidentified as bladder malignancy. Crucially, dynamic cystoscopic features (protrusion upon bladder emptiness and retraction during filling) and recurrent urinary flocculent material emerged as pivotal yet overlooked clues to enterovesical fistula formation.^[9]^ To our knowledge, this represents the first documented case of a small bowel diverticulum simulating recurrent bladder cancer through endophytic growth into the bladder lumen, ultimately confirmed as vesico-enteric fistula (VIF) via combined urological and gastrointestinal surgical intervention. This report aims to: detail the clinical trajectory and critical intraoperative findings; analyze systemic causes of prolonged misdiagnosis, including technical and cognitive factors; propose strategies to enhance early detection (e.g., leveraging dynamic imaging signs and multidisciplinary team [MDT] collaboration); highlight the imperative for considering intestinal pathologies in “atypical bladder tumor” cases.^[9]^

## 2. Case report

This study was approved by the Institutional Review Board of Binzhou Medical University Hospital (Approval No. YT-2024-CS-008). The patient provided written informed consent for the publication of this case report and any accompanying images.

### 2.1. Demographics and baseline characteristics

A 35-year-old Chinese female with permanent residency in the United States presented for initial urological evaluation in December 2022. Her medical history was significant only for an appendectomy performed at an unspecified prior date. She denied any chronic illnesses (hypertension, diabetes, and coronary artery disease), infectious diseases (viral hepatitis and tuberculosis), or prior transfusion history. The patient was nulliparous with regular menstrual cycles (menarche at age 13, cycle 28–30 days). Family history was noncontributory for malignancies or similar conditions. She had no history of smoking, alcohol consumption, or occupational exposure to toxins.

### 2.2. Chronological clinical course and management

#### 2.2.1. Phase 1: initial misdiagnosis as malignant bladder tumor (U.S. Hospital)

*December 2022*: the patient developed acute urinary frequency, urgency, dysuria, and persistent right lower quadrant (RLQ) abdominal pain. Empirical antibiotic therapy (agent/duration unspecified) failed to alleviate symptoms.

*January 2023*: diagnostic cystoscopy revealed a bladder mass highly suspicious for urothelial carcinoma. The presence of this mass, in conjunction with her irritative lower urinary tract symptoms, constituted the standard and primary indication for performing the initial transurethral resection of bladder tumor (TURBT) for diagnostic and therapeutic purposes.

*March 8, 2023*: under general anesthesia, standard TURBT was performed. The operative note described resection of a “friable, papillary lesion” on the right lateral wall.

*Pathology report (institutional summary*): “Malignant epithelial neoplasm consistent with high-grade urothelial carcinoma.” It is important to note that the patient was only able to obtain a summary report from the U.S. institution; the full histopathology report and original slides were not available for our review. This represents a limitation in the initial diagnostic data.

*Postoperative protocol deviation*: adjuvant intravesical chemotherapy or Bacillus Calmette-Guérin therapy was not administered, contrary to standard guidelines for high-grade disease.

*Persistent symptoms*: the patient reported new-onset expulsion of flocculent, particulate matter during micturition within 2 weeks postoperatively. Repeat cystoscopy at 6 weeks showed “residual/recurrent tumor.”

#### 2.2.2. Phase 2: re-resection with benign pathology (Yantai Yuhuangding Hospital)

*September 19, 2023*: the patient sought further management in China due to unresolved symptoms.

*Preoperative workup*: urinalysis: pyuria (white blood cell > 50/high-power field), no hematuria. *Urine cytology*: atypical cells, inconclusive for malignancy. Renal and bladder ultrasound: focal bladder wall thickening (right posterolateral), no hydronephrosis.

*September 21, 2023*: repeat TURBT performed under general anesthesia:

*Cystoscopic findings*: a 2.0 × 1.0 cm area of necrotic, follicular-appearing tissue at the prior resection site (right lateral wall). No other lesions.

*Surgical technique*: resection to the deep muscularis propria using a 24Fr Olympus resectoscope. Hemostasis achieved with rollerball coagulation. A 22Fr Foley catheter was placed postoperatively.

*Histopathology report*: “Fragments of benign urothelial tissue with extensive ulceration, granulation tissue formation, and chronic inflammation. No evidence of malignancy.”

*Adjuvant therapy*: weekly intravesical instillation of 30 mg pirarubicin initiated for 6 weeks (exact regimen not detailed in records).

#### 2.2.3. Phase 3: life-threatening hemorrhage and bladder rupture (Binzhou Medical University Hospital Yantai)

*October 13, 2023*: acute presentation with massive gross hematuria, bladder distension, and suprapubic pain following deliberate urine retention.

*Emergency imaging*: ultrasound: “Bladder lumen filled with an 8.2 × 7.1 cm heterogeneous, nonvascularized mass-like echogenicity consistent with clot. Focal bladder wall discontinuity (right dome).” *CT cystogram*: deferred due to hemodynamic instability (BP 85/50 mm Hg, hemoglobin drop from 12.8–7.4 g/dL).

Surgical intervention (sequential approach):

Step 1: Transurethral clot evacuation

Cystoscopy: “Complete obliteration of visual field by fresh blood clots.”

Partial clot removal via Ellik evacuator (unsuccessful in controlling bleeding).

Step 2: Emergent open exploration

Midline infraumbilical incision (10 cm).

*Key findings*: intraperitoneal bladder rupture (1.5 cm defect at dome). Two arterial bleeders at prior resection bed (right lateral wall). 500 mL intravesical and 300 mL intraperitoneal clots. (It is crucial to contextualize this emergency procedure. The primary surgical mandate was life-saving hemorrhage control and repair of the acute bladder rupture in an unstable patient. The operative field was significantly compromised by massive hematuria, organized clots, and dense inflammatory adhesions from previous surgeries. Under these exigent circumstances, a meticulous exploration for a potential fistulous tract, which can be subtle, was not feasible and carried a high risk of iatrogenic injury.)

*Repair*: ligation of bleeders with 3-0 Vicryl. Bladder rupture closure: two-layer repair (mucosa: 2-0 Monocryl continuous; seromuscular: 3-0 Silk Lembert). Suprapubic tube (22Fr Malecot) + urethral catheter placement.

Pathology (clot analysis): “Organized fibrin with embedded inflammatory cells—no malignant cells identified.”

#### 2.2.4. Phase 4: definitive diagnosis and multidisciplinary management (same hospital)

*August 9, 2024*: as part of the long-term surveillance plan for her complex bladder history (including prior resections and bladder rupture repair), the patient underwent a scheduled surveillance cystoscopy.

*Dynamic cystoscopic sign*: A 0.8 × 0.8 cm broad-based, cauliflower-like lesion on the right posterior wall.

*Pathognomonic observation*: Protrusion through a mucosal defect when bladder emptied (50 mL), complete retraction at capacity (250 mL) (“This dynamic protrusion-retraction phenomenon was meticulously documented in the operative notes and served as the pivotal intraoperative clue prompting the suspicion of an enterovesical fistula.”)

**Figure 1.**
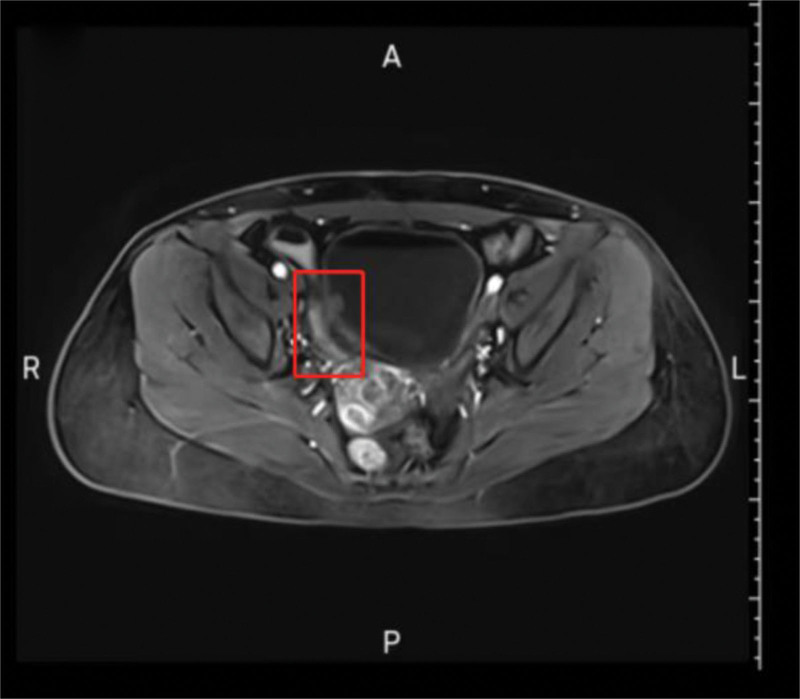
Preoperative pelvic MRI (T2-weighted image) showing abnormal signal at the right posterolateral bladder wall (arrow) with extraluminal tract to ileum. MRI = magnetic resonance imaging.

Preoperative magnetic resonance imaging (MRI) (August 10, 2024; Fig. 1): T2-weighted imaging: “Extraluminal soft-tissue tract connecting a 3 cm sac-like structure (ileum) to the right posterolateral bladder wall. Fistula tract diameter: 8 mm.” Diffusion-weighted imaging/apparent diffusion coefficient: no restricted diffusion to suggest malignancy.

*August 11, 2024*: combined procedure.

##### 2.2.4.1. Part A: diagnostic/therapeutic TURBT

Lesion resection: villous architecture with firm attachment to a 0.5 × 0.5 cm deep wall defect. Critical intraoperative event: after fulguration of the base, yellow, turbid fluid with gas bubbles extruded retrograde through the defect. Simultaneous expulsion of flocculent material per urethra. Diagnosis: suspected enterovesical fistula.

##### 2.2.4.2. Part B: laparoscopic exploration and repair (GI surgery collaboration)

**Figure 2.**
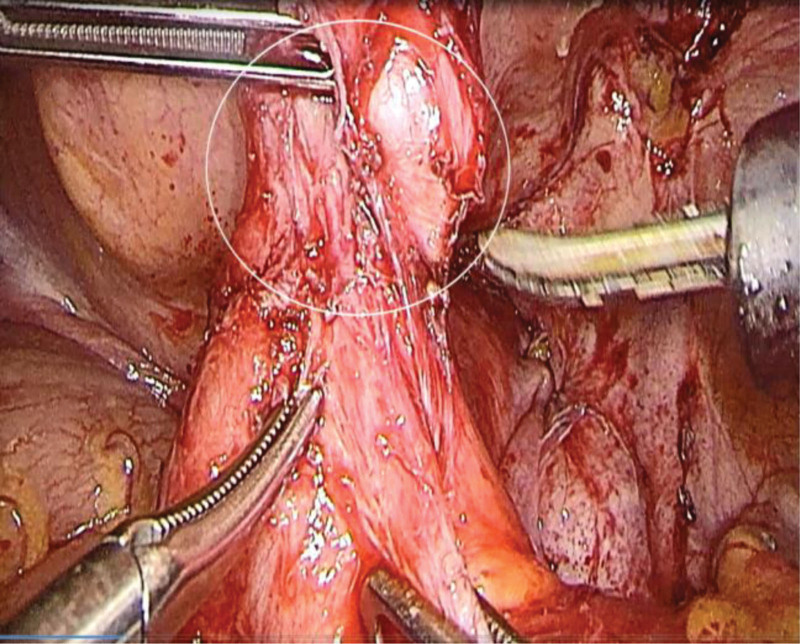
Laparoscopic view of the ileal diverticulum adherent to the bladder.

Port placement: 5 ports (umbilical 10 mm, RLQ 12 mm, others 5 mm). Adhesiolysis: dense omental and ileopelvic adhesions (from prior laparotomy). Pathognomonic findings: terminal ileal diverticulum (3 × 3 × 2.5 cm) 20 cm proximal to ileocecal valve (Fig. 2). Fistulous tract (10 mm diameter) to bladder dome. No malignancy, Crohn disease, or foreign body.

### 2.3. Surgical sequence

**Figure 3.**
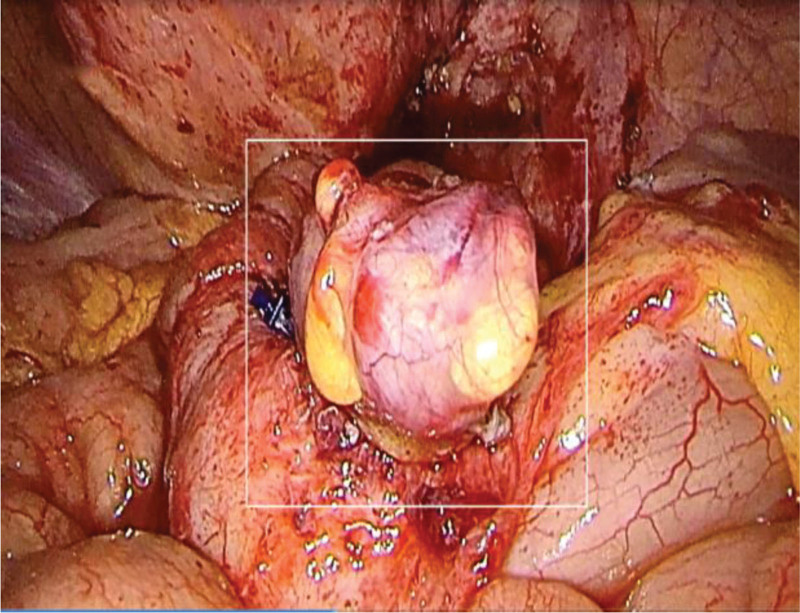
Resected diverticulum specimen with fistula orifice.

Diverticulectomy: en bloc resection with 60 mm linear stapler (Ethicon Echelon) (Fig. 3). Fistula repair: bladder defect closed in 2 layers (3-0 V-Loc running suture; omental flap interposition). Adhesiolysis: ultrasonic dissection (Harmonic Ace). Drain: 19Fr Blake drain pelvis. Intraoperative consultation: frozen section of bladder edge: “Chronic inflammation with glandular metaplasia—negative for malignancy.”

### 2.4. Pathological and immunohistochemical correlation

#### 2.4.1. Specimen 1: bladder resection (fourth TURBT) (Fig. 4)

Gross: fragmented tan tissue (aggregate 1.2 cm). Microscopy: ulcerated mucosa with intense chronic inflammation (lymphoplasmacytic). Nephrogenic adenoma-like hyperplasia: tubulocystic structures lined by cuboidal epithelium. Foci of colonic metaplasia: mucin-secreting glands. Immunohistochemistry (Table 1).

**Table 1:** Immunohistochemistry.

Marker	Metaplastic epithelium	Intestinal epithelium
PAX-8	Positive	Negative
CK7	Positive	Negative
CK20	Negative	Positive
GATA3	Negative	Negative
Ki-67	<1%	<1%

**Figure 4.**
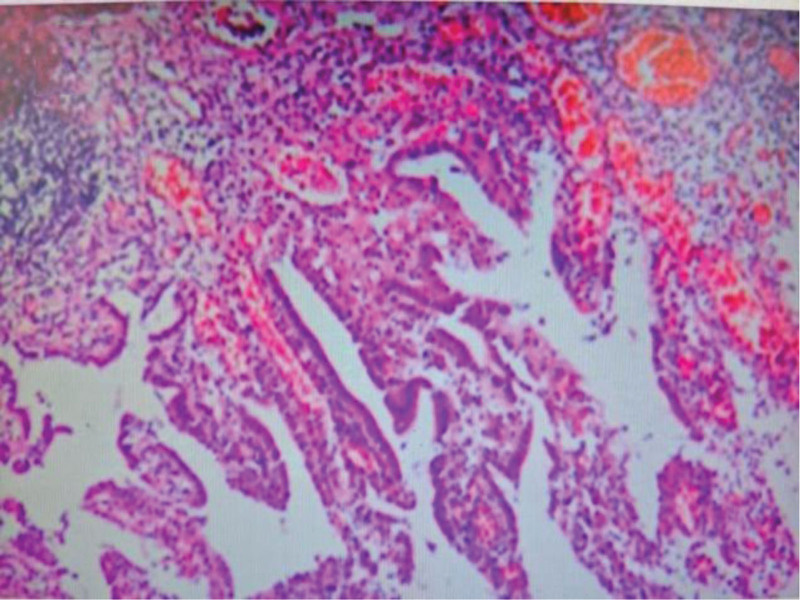
Histopathology of bladder mucosa: nephrogenic adenoma-like hyperplasia (H&E, ×200).

#### 2.4.2. Specimen 2: ileal diverticulum

Gross: sac-like structure with 10 mm communication orifice. Microscopy: full-thickness ileal wall with herniated mucosa/submucosa. No muscularis propria in diverticular wall. Mucosa: chronic active ileitis with erosions, no granulomas or dysplasia.

### 2.5. Postoperative recovery and follow-up

Days 1 to 3: urethral catheter output: clear urine, no particulate matter. Pelvic drain: serosanguinous (50 mL/day). Day 4: normal bowel function resumed. Day 7: cystogram: no contrast extravasation. Catheter/drain removed. 3-Month follow-up (November 2024): cystoscopy: healed fistula site, no recurrence. CT enterography: no residual diverticulum or fistula.

*Final diagnosis*: complicated acquired ileal diverticulum with VIF.

## 3. Discussion

### 3.1. Clinical characteristics and diagnostic dilemmas of small intestinal diverticula

Small intestinal diverticula affect 1% to 5% of adults,^[10]^ with diagnostic challenges arising from 3 inherent characteristics: clinical insidiousness: 70% to 85% remain asymptomatic lifelong; complications trigger clinical presentation.^[11,12]^ This case’s initial symptoms (urinary frequency, urgency, and RLQ pain) overlapped significantly with bladder cancer, while the critical clue of flocculent urine (later confirmed as enteric content via fistula) was misattributed to “postoperative necrotic tissue,” delaying intestinal pathology evaluation. Imaging limitations: cystoscopy detects transmural/extramural penetration in only 30% to 40% of cases.^[13,14]^ The first 3 cystoscopies here reported “tumor-like lesions” but missed the dynamic filling sign (protrusion when empty/retraction when full) (documented only preoperatively during the fourth episode and pathognomonic for diverticular intrusion). MRI showed abnormal signal at the right posterior bladder wall (Fig. 1) but excretory-phase dynamic scanning was omitted, missing fistula contrast flow and leading to tumor misdiagnosis. Pathological misinterpretation risks: the initial TURBT reported “malignancy” (lacking full documentation), conflicting with the second pathology’s “benign” result. Final pathology revealed the truth: bladder mucosa showed nephrogenic adenoma-like hyperplasia (PAX-8+/CK7+) and intestinal metaplasia (CK20+), both reactive to chronic inflammation rather than true neoplasia.

### 3.2. Multidimensional analysis of misdiagnosis mechanisms

#### 3.2.1. Cognitive bias: anchoring effect in clinical decision-making chain reaction from initial misdiagnosis

The first “malignant” diagnosis (despite missing pathology) triggered anchoring bias,^[15,16]^ causing subsequent interventions to prioritize tumor recurrence/residual disease over rare etiologies. Studies show >60% of misdiagnosed cases stem from persistent initial diagnostic labels. Ignoring contradictory evidence: the second TURBT pathology was “benign,” yet clinicians administered pirarubicin instillations (reflecting confirmation bias: selectively accepting cystoscopic “mass” appearance while dismissing counterevidence [benign pathology and flocculent urine]).

#### 3.2.2. Technical limitations: diagnostic blind spots and errors

Inherent cystoscopy deficiencies: visual blind zones: the first 3 procedures failed to systematically examine the bladder dome, potentially missing early fistula signs. Interference factors: massive hemorrhage during the third surgery (“clots filling bladder”) obscured visualization of potential fistulas. Imaging deficiencies: pelvic MRI used plain scans only, omitting multiphasic contrast enhancement or CT cystography (>90% sensitive for fistulas). No oral tracer tests (e.g., charcoal challenge) to confirm enteric material in urine.

#### 3.2.3. Pathological diagnostic traps: evolution from “Malignancy” to “Metaplasia”

The initial “malignant” diagnosis lacked substantiation (missing report), while evolving pathology revealed: nephrogenic adenoma: present in 60% to 80% of chronic bladder irritation (e.g., stones and fistulas), often mimicking adenocarcinoma. Intestinal metaplasia: adaptive mucosal change near fistulas; CK20+ expression matched diverticular mucosa (Fig. 4).This explains pathological discrepancies across 4 specimens (inflammatory responses fluctuated with fistula activity, not tumor biology).

### 3.3. Critical reflections on treatment: turning points and decision optimization in 4 surgeries (Table 2)

#### 3.3.1. Core controversy: was avoiding bowel exploration justified during the third surgery?

Supporting view: life-threatening factors (rupture/bleeding) required prioritization; prior surgery ↑ bowel injury risk. Opposing view: bladder dome rupture + flocculent urine history should prompt limited exploration (e.g., ileal palpation).

**Table 2:** Critical reflections on treatment.

Surgery	Key decision	Reflection and optimization
First	No instillation post-“malignancy”	Violated EAU NMIBC guidelines^[5]^; timely instillation might mitigate inflammation
Third	Prioritized TUR for hemorrhage	Active bleeding with shock warrants immediate open exploration; delayed laparotomy ↑ bladder rupture risk

EAU = European Association of Urology, NMIBC = Non-Muscle-Invasive Bladder Cancer.

#### 3.3.2. Evaluation of etiology: primary diverticulum versus iatrogenic injury

Anatomic and temporal discordance: the fistula from the ileal diverticulum was definitively identified at the right posterolateral bladder wall during the final laparoscopic procedure. In contrast, the bladder rupture addressed during the third emergency surgery was located at the bladder dome. These are 2 distinct anatomical sites. A perforation on the dome is unlikely to directly cause a fistulous tract to form on the posterolateral wall without evidence of a contiguous inflammatory process, which was not observed.

Histopathological evidence of chronicity: the resected ileal diverticulum exhibited microscopic features of “chronic active ileitis with erosions.” This indicates a long-standing, indolent inflammatory process within the diverticulum itself, which is the hallmark of a primary diverticular disease process leading to transmural inflammation and fistulization. This is inconsistent with a fresh iatrogenic perforation.

Symptom timeline: the patient reported the new onset of expelling flocculent material in her urine within 2 weeks after the first TURBT. This timeline strongly suggests that a communication between the inflamed diverticulum and the bladder already existed or was in its final stages of formation at the time of the initial procedure. It is less plausible that a TURBT perforation would instantaneously create a mature fistula capable of passing enteric content within such a short period.

While we cannot entirely rule out that the repetitive transurethral resections and associated pelvic inflammation may have exacerbated the fistulous process or contributed to adhesion formation, the cumulative evidence (particularly the chronic histology and the early presentation of pathognomonic symptoms) more robustly supports the sequence of a primary complicated small bowel diverticulum eroding into the bladder.

### 3.4. Literature comparison and case uniqueness

#### 3.4.1. Clinical features of diverticulum-associated VIF: literature comparison (Table 3)

Systematic review (PubMed, 2000–2025) of key features: table: reported cases of small bowel diverticulum-induced VIF.

**Table 3:** Literature comparison.

Study	Age/sex	Preoperative misdiagnosis	Diagnostic method	Dynamic sign	Surgical approach
Zhang et al.^[10]^	42/M	Crohn disease	Laparotomy	Not recorded	Open resection
Sulaiman et al.^[3]^	29/M	Diverticular bleeding	Reoperation	Absent	Open resection
Coutinho et al.^[4]^	76/M	Bowel obstruction	CT + surgery	Absent	Laparoscopic
This case	35/F	Bladder cancer	Intraop. fistula sign	Present	Laparoscopic

#### 3.4.2. Three unique aspects of this case

Misdiagnosis frequency/severity: four surgeries (malignant → benign → hemorrhage → tumor recurrence) (longest diagnostic chain in literature). Specific dynamic sign: bladder emptiness-triggered protrusion/filling-induced retraction (previously reported in only 2 bladder diverticulum cases); first documentation in small bowel diverticulum. Comprehensive pathological evolution: full pathological documentation from “malignancy” to “inflammatory metaplasia,” revealing cytological basis of misdiagnosis.

### 3.5. Clinical implications and improvement strategies

#### 3.5.1. Diagnostic strategy optimization

**Figure 5.**
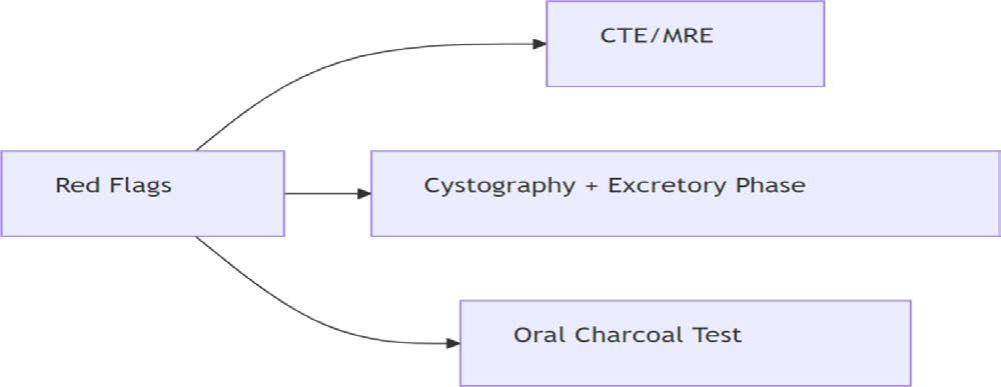
Flowchart of “Red Flag” response protocol.

“Red Flag” response protocol (Fig. 5): for recurrent “bladder tumors” with any of: flocculent urine; pneumaturia; fecaluria; dynamic filling sign,immediately initiate.

### 3.6. Clinical implications and improvement directions

#### 3.6.1. Diagnostic strategy optimization

Enhance recognition of rare diseases: incorporate small bowel diverticulum into abdominal disorder differential diagnoses, particularly for recurrent “bladder tumors” with atypical features (e.g., flocculent urine). Establish MDT pathways: implement mandatory urology–gastrointestinal surgery–radiology collaboration for complex cases after initial misdiagnosis. Rationalize invasive diagnostics: prioritize diagnostic laparoscopy when noninvasive imaging fails, especially with fistula suspicion.

#### 3.6.2. Technical application principles

Standardize cystoscopic practice: document bladder filling state (0 mL/300 mL) for all recurrent lesions. Biopsy submucosal layers near defects to detect fistulous tracts. Intraoperative vigilance: perform dynamic assessment under varying bladder filling states. Test defect sites with methylene blue instillation if fistula suspected.

#### 3.6.3. Healthcare quality enhancement

Rare disease competency training: integrate enterovesical fistula modules into urology board exams. Develop simulation training for dynamic cystoscopic sign recognition. Decision-support systems: embed AI-based diagnostic checklists for “red flag” symptoms (flocculent urine and pneumaturia).

### 3.7. Concluding statement

This report illuminates how small bowel diverticula may mimic bladder malignancies, culminating in our patient’s 4-surgeon odyssey. We advocate for: systematic rare disease education, protocolized MDT integration, and technology-enhanced decision support. These measures will enhance diagnostic precision, reduce unnecessary interventions, and optimize patient-centered outcomes through reduced misdiagnosis-related harm.

## Author contributions

**Conceptualization:** Jinting Wu, Yinglei Wang.

**Data curation:** Jinting Wu, Yinglei Wang.

**Formal analysis:** Yang Zhao.

**Funding acquisition:** Yang Zhao.

**Methodology:** Guangming Shan.

**Project administration:** Guangming Shan.

**Software:** Dongbing Zheng.

**Supervision:** Dongbing Zheng.
